# PPARγ Regulates Trophoblast Proliferation and Promotes Labyrinthine Trilineage Differentiation

**DOI:** 10.1371/journal.pone.0008055

**Published:** 2009-11-30

**Authors:** Mana M. Parast, Heather Yu, Aleksandar Ciric, Mark W. Salata, Vannessa Davis, David S. Milstone

**Affiliations:** 1 Department of Pathology, University of California San Diego, La Jolla, California, United States of America; 2 Center for Excellence in Vascular Biology, Vascular Research Division, Department of Pathology, Brigham and Women's Hospital, Boston, Massachusetts, United States of America; Cincinnati Children's Research Foundation, United States of America

## Abstract

**Background:**

Abnormal trophoblast differentiation and function is the basis of many placenta-based pregnancy disorders, including pre-eclampsia and fetal growth restriction. PPARγ, a ligand-activated nuclear receptor, plays essential roles in placental development; null murine embryos die at midgestation due to abnormalities in all placental layers, in particular, small labyrinth and expanded giant cell layer. Previous studies have focused mostly on the role of PPARγ in trophoblast invasion. Based on the previously reported role of PPARγ in preadipocyte differentiation, we hypothesized that PPARγ also plays a pivotal role in trophoblast differentiation. To test this hypothesis, we report derivation of wild-type and PPARγ-null trophoblast stem (TS) cells.

**Methodology/Principal Findings:**

PPARγ-null TS cells showed defects in both proliferation and differentiation, specifically into labyrinthine trophoblast. Detailed marker analysis and functional studies revealed reduced differentiation of all three labyrinthine lineages, and enhanced giant cell differentiation, particularly the invasive subtypes. In addition, rosiglitazone, a specific PPARγ agonist, reduced giant cell differentiation, while inducing Gcm1, a key regulator in labyrinth. Finally, reintroducing PPARγ into null TS cells, using an adenovirus, normalized invasion and partially reversed defective labyrinthine differentiation, as assessed both by morphology and marker analysis.

**Conclusions/Significance:**

In addition to regulating trophoblast invasion, PPARγ plays a predominant role in differentiation of labyrinthine trophoblast lineages, which, along with fetal endothelium, form the vascular exchange interface with maternal blood. Elucidating cellular and molecular mechanisms mediating PPARγ action will help determine if modulating PPARγ activity, for which clinical pharmacologic agonists already exist, might modify the course of pregnancy disorders associated with placental dysfunction.

## Introduction

Placental dysfunction, caused by abnormalities in trophoblast differentiation and function, is associated with many pregnancy disorders, including intrauterine growth restriction and preeclampsia [reviewed in 1]. Peroxisome proliferator-activated receptors (PPARs), which are transcription factors and members of the ligand-activated nuclear hormone receptor superfamily, play major roles in diverse aspects of energy metabolism, inflammation, and development [2]–[5]. Following ligand binding, PPARs form heterodimers with retinoid X receptors (RXRs), and bind to PPAR-response elements (PPREs) of target genes to activate transcription [2]. PPARγ has received much attention with regard to its role in adipogenesis and energy metabolism, including its highly efficacious synthetic ligands, the thiazolidinediones, which are routinely used clinically for treatment of type 2 diabetes [3], [4]. However, genetically null mice revealed a surprising role for PPARγ: namely its essential role in placental development [6], [7]. Since this discovery, additional studies have focused primarily on the effect of PPARγ ligands in human trophoblast, revealing effects on both syncytiotrophoblast function [8] and trophoblast invasion [9]. Further studies in mice have identified some transcriptional targets of PPARγ in trophoblast [10] and shown that treatment of pregnant mothers with PPARγ agonists led to fetal and placental growth restriction in a PPARγ-dependent manner [11]. However, specific cellular and molecular mechanisms by which this important transcription factor exerts its predominate role in trophoblast differentiation and placental development have not been elucidated.

Given the essential role of PPARγ in adipocyte differentiation [12], [13], and the abnormalities observed in specific layers of PPARγ-null placentas, we hypothesized that PPARγ also regulates critical aspects of normal trophoblast differentiation. In order to test this hypothesis, we generated both wild-type (WT) and PPARγ-null trophoblast stem (TS) cells, from E3.5 murine blastocysts using previously published methods [14]. Murine TS cells are an invaluable tool for characterizing the role of specific genes in trophoblast differentiation and function [15]–[17]. In this study, we characterized cellular properties and the differentiation capacity of PPARγ-null TS cells and found a predominate role for this protein in both trophoblast proliferation and labyrinthine trophoblast differentiation.

## Results

### PPARγ^−/−^ TS Cells Grow Slower than Their Wild-Type Counterparts

**Figure 1:**
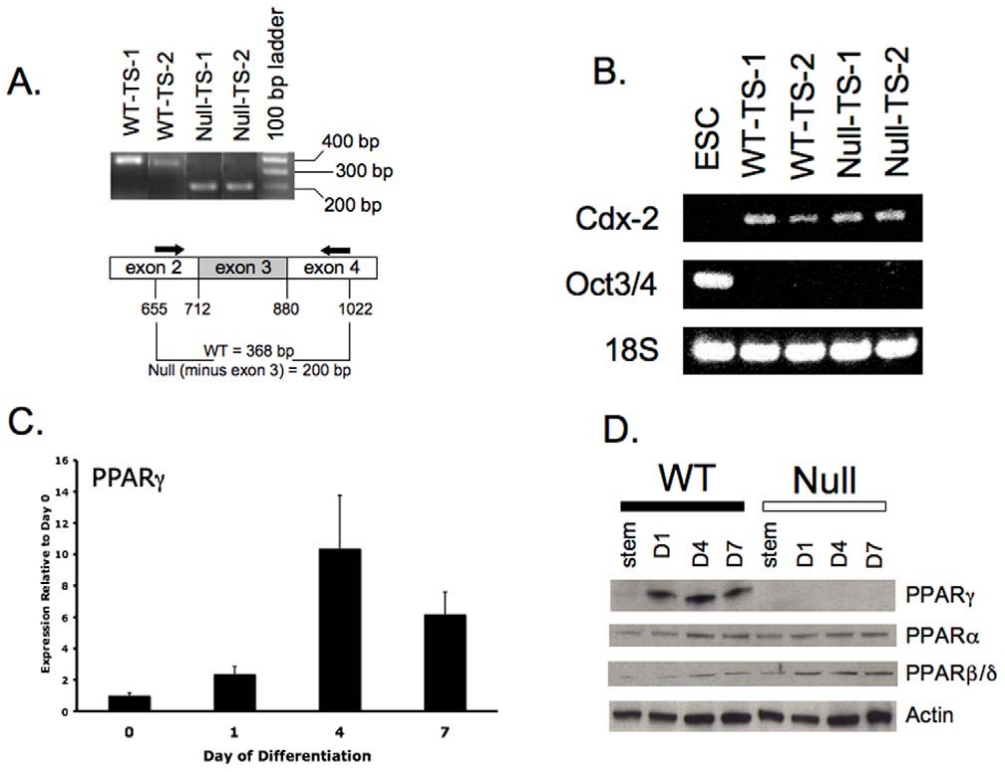
Derivation of PPARγ^+/+^ and PPARγ^−/−^ TS cells. A) Confirmation of wild-type and null genotypes, using RT-PCR with primers flanking the deleted exon 3 in the PPARγ1 gene. Arrows indicate the position of primers used for PCR. B) Both WT and null TS cells express the trophectoderm marker Cdx2, but not ICM-specific Oct3/4, which is limited to ES cells. C) PPARγ mRNA expression in WT TS cells peaks at day 4 of differentiation, based on quantitative RT-PCR. D) Western blot for PPARγ, PPARα, and PPARβ/δ, in WT and null TS cells.

PPARγ^+/+^ and PPARγ^−/−^ TS cells were generated from E3.5 blastocysts from two heterozygous matings. Two PPARγ^+/+^, one PPARγ**^+/−^**, and three PPARγ**^−/−^** TS cell lines were obtained from 14 explanted blastocysts (43% overall yield). Both PPARγ^+/+^ and two PPARγ**^−/−^** TS cell lines were chosen for further study. Both wild-type (WT) lines were analyzed in most experiments and did not show statistically significant differences. Results from one WT line are shown for simplicity. TS cell genotypes, determined by PCR of genomic DNA, were confirmed at the phenotypic expression level using RT-PCR of total cell RNA with primers surrounding exon 3, which is deleted in the null allele (Figure 1A), and by Western blot (Figure 1D). Both WT and null lines expressed markers of trophectoderm (Cdx-2, Eomes, Errβ; see Table 1 for definitions of gene abbreviations), and lacked inner cell mass (ICM)/embryonic stem (ES) cell markers (Oct3/4) (Figure 1B, and data not shown). In WT TS cell cultures, PPARγ mRNA and protein were detected at very low levels in the undifferentiated state, but were strongly upregulated upon differentiation, with maximal expression observed at day 4 (Figures 1C and 1D). In contrast PPARγ protein was not detected in PPARγ**^−/−^** TS cell cultures at any stage of differentiation (Figure 1D). In addition, while levels of PPARα were comparable between WT and null TS cells, PPARβ/δ was upregulated (1.5–2.5 fold by densitometry, normalized to βactin expression) in differentiated null cells compared to PPARγ^+/+^ cells (Figure 1D); these changes in PPARα and PPARβ/δ protein levels were reflective of changes in their respective mRNA levels (data not shown).

**Table 1:** List of primers.

Gene abbreviation	Gene Name	Primer sequence
Errβ	Estrogen-related receptor-β	F 5′ GGGAGCTTGTGTTCCTCATC 3′
		R 5′ CTACCAGGCGAGAGTGTTCC 3′
Cdx2	Caudal type homeobox-2	F 5′ CCAGCTCTTTGCCTCTCTGT 3′
		R 5′ TGCCTCTGGCTCCTGTAGTT 3′
Oct3/4 (Pou5f1)	POU domain transcription factor-1	F 5′ GCCGTGAAGTTGGAGAAGGT 3′
		R 5′ GAAGCGACAGATGGTGGTCT 3′
Pl-I	Placental lactogen-1	F 5′ TGGTGTCAAGCCTACTCCTTT 3′
		R 5′ CAGGGGAAGTGTTCTGTCTGT 3′
Pl-II	Placental lactogen-2	F 5′ CCAACGTGTGATTGTGGTGT 3′
		R 5′ TCTTCCGATGTTGTCTGGTG 3′
Tpbpa (4311)	Trophoblast specific protein alpha	F 5′ CGGAAGGCTCCAACATAGAA 3′
		R 5′ TCAAATTCAGGGTCATCAACAA 3′
synA	Syncytin-A	F 5′ CCCTTGTTCCTCTGCCTACTC 3′
		R 5′ TCATGGGTGTCTCTGTCCAA 3′
Gcm1	Glial cells missing-1	F 5′ AACACCAACAACCACAACTCC 3′
		R 5′ CAGCTTTTCCTCTGCTGCTT 3′
muc1	Mucin 1	F 5′ TTGGTTGCTTTGGCTATCGT 3′
		R 5′ TTACCTGCCGAAACCTCCTC 3′
Tfeb	Transcription factor EB	F 5′ AACAAAGGCACCATCCTCAA 3′
		R 5′ CAGCTCGGCCATATTCACAC 3′
Ctsq	Cathepsin Q	F 5′ AACTAAAGGCCCCATTGCTAC 3′
		R 5′ CAATCCCCATCGTCTACCC 3′
Plf	Proliferin	F 5′ TGAGGAATGGTCGTTGCTTT 3′
		R 5′ TCTCATGGGGCTTTTGTCTC 3′
PPARγ (qRT-PCR)	Peroxisome proliferator activated receptor gamma	F 5′ GACAGGAAAGACAACGGACAA 3′
		R 5′ AAACTGGCACCCTTGAAAAA 3′
PPARγ (genotyping)	Peroxisome proliferator activated receptor gamma	F 5′ GAGCTGACCCAATGGTTGCTGATT 3′
		R 5′ GCATTATGAGACATCCCC 3′
PPARα	Peroxisome proliferator activated receptor alpha	F 5′ ACACCCTCTCTCCAGCTTCC 3′
		R 5′ AATCTTGCAGCTCCGATCAC 3′
PPARβ/δ	Peroxisome proliferator activated receptor delta	F 5′ CGGGAAGAGGAGAAAGAGGA 3′
		R 5′ GTGGACCCCGTAGTGGAAG 3′
18S	18S ribosomal subunit	F 5′ CGCGGTTCTATTTTGTTGGT 3′
		R 5′ AACCTCCGACTTTCGTTCTTG 3′

**Figure 2:**
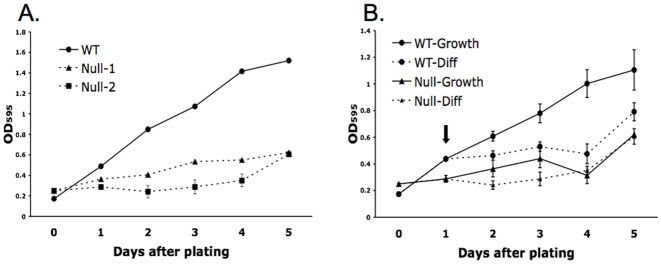
PPARγ^−/−^ TS cells grow slower than their WT counterparts. MTT assay comparing A) WT and null TS cell growth over a 5 day period in TSMFH medium (note: standard deviations are too small to observe for WT and Null-1 data) or B) WT and null TS cell growth in TSMFH (growth) vs. TS (differentiation) medium. Arrow in B shows the point at which the indicated cultures were switched from TSMFH to TS medium.

**Figure 3:**
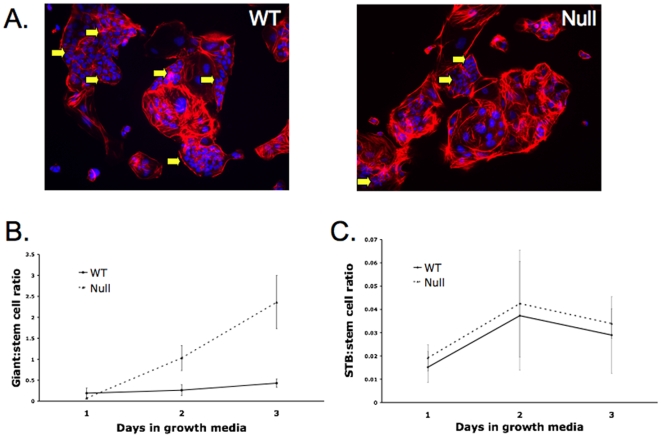
PPARγ^−/−^ TS cells prematurely differentiate into giant cells. A) PPARγ^−/−^ TS cells show fewer undifferentiated colonies (arrows) than WT TS cells when cultured in TSMFH medium. Cells are double-stained with phalloidin (red) and DAPI (blue). B and C) Quantitation of trophoblast giant cell (TGC) (B) and syncytiotrophoblast (STB) (C) differentiation, assessed by morphology as in panel A and described in the legend to Figure 4, in WT and null TS cells grown for 3 days in TSMFH media. 200–300 cells were counted for each time point in each of three independent experiments.

Morphologically, both WT and null cells formed typical undifferentiated colonies when grown in TSMFH media (standard TS cell growth media; see Materials and Methods), and differentiated to form trophoblast giant cells (TGC's) when switched to TS media (differentiation media: withdrawal of conditioned media, FGF4 and heparin) (see below). However, both PPARγ^−/−^ lines in TSMFH media grew more slowly (1.5–3-fold) than WT TS cells (Figure 2A) such that expansion of PPARγ**^−/−^**, but not PPARγ^+/+^ cultures was similar under growth and differentiation conditions (Figure 2B). To examine stem cells more directly, and to assess whether slow overall growth in PPARγ**^−/−^** cultures was due to accumulation and carry-over of post-mitotic giant cells during passage, we lightly trypsinized both PPARγ^+/+^ and PPARγ**^−/−^** cultures (selectively releasing undifferentiated cells), plated the resultant nonadherent, stem cell enriched populations at equivalent densities (10^5^ cells per well in a 6-well plate) and assessed growth and morphologic differentiation in TSMFH media over a 3-day period (Figure 3). The ratio of giant cells to undifferentiated (stem) cells was initially similar in PPARγ^+/+^ and PPARγ**^−/−^** cultures, but increased significantly over time only in the latter (Figure 3B). This indicates that absence of PPARγ leads to premature differentiation of TS cells into, and not carry-over of, giant cells even in the presence of MEF-conditioned medium, FGF4 and heparin. Differentiation of syncytiotrophoblast under these conditions was negligible in both PPARγ^+/+^ and PPARγ**^−/−^** cultures, with no significant difference between the two cell types (Figure 3C).

### PPARγ^−/−^ TS Cells Exhibit Altered Trophoblast Lineage Differentiation *In Vitro*

**Figure 4:**
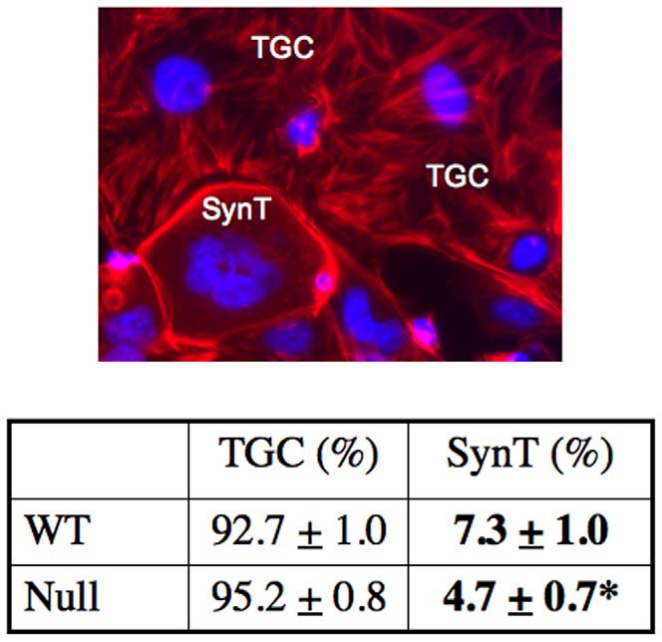
PPARγ^−/−^ TS cells show decreased syncytiotrophoblastic differentiation. Syncytiotrophoblast (synT) are defined by the presence of three or more nuclei (identified by blue DAPI fluorescence) with relatively few actin stress fibers (identified by red phalloidin fluorescence), while giant cells (TGC) characteristically contained one-to-two nuclei with highly organized actin stress fibers. The results are from three independent experiments; for each, 550–600 cells were counted (*P = 0.0227 compared to WT).

We differentiated both WT and null cells for 7-days and assessed labyrinthine (syncytiotrophoblastic) and invasive trophoblast/giant cell differentiation both by morphology and qRT-PCR. Morphologic differentiation was assessed by double staining with phalloidin and DAPI to distinguish giant cells, with one or two large nuclei (each at least double the size of a stem cell nucleus) from multi-nucleated syncytiotrophoblast (three or more nuclei each equivalent in size to stem cell nuclei). Morphologic analyses revealed that syncytiotrophoblasts were significantly less abundant in differentiated PPARγ**^−/−^** than in PPARγ**^+/+^** TS cells (4.7±0.7% vs. 7.3±1.0%, respectively, *P* = 0.023) (Figure 4).

**Figure 5:**
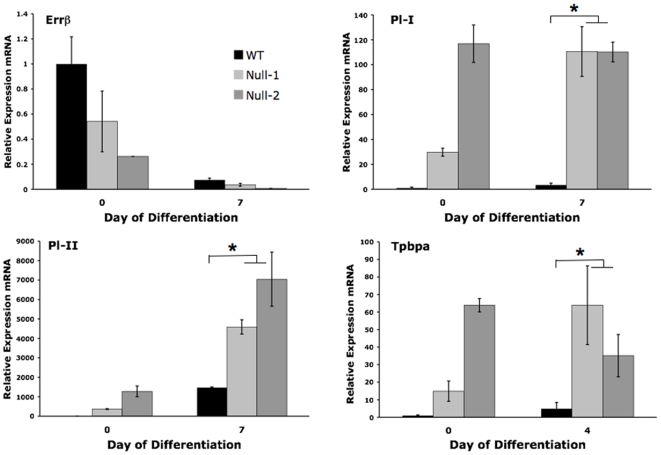
PPARγ^−/−^ TS cells show enhanced spongiotrophoblast and giant cell marker expression by quantitative RT-PCR. Note lower expression of undifferentiated marker, Errβ, in PPARγ^−/−^ TS cells under growth conditions (day 0); along with increased spongiotrophoblast and giant cell markers, this further emphasizes their premature differentiation under growth conditions. All values are expressed relative to undifferentiated WT TS cells. *indicates statistically significant (P≤0.05) alteration in marker expression in null cells, compared to WT cells.

**Figure 6:**
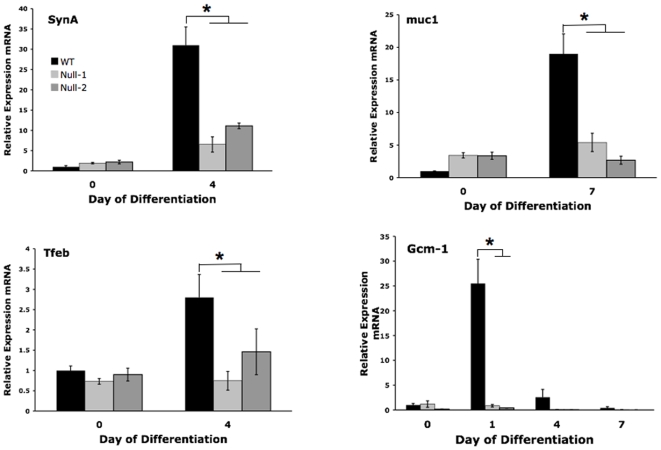
PPARγ^−/−^ TS cells show decreased labyrinthine markers by quantitative RT-PCR. Note the dramatic decrease in Gcm1, an essential regulator of labyrinthine differentiation. All values are expressed relative to undifferentiated WT TS cells. *indicates statistically significant (P≤0.05) alteration in marker expression in null cells, compared to WT cells.

In preliminary experiments, differentiating WT and PPARγ^−/−^ cells were analyzed daily for 7 days by qRT-PCR to determine the time of maximal expression of each lineage marker. The results were similar to previously published data, indicating maximal expression of Gcm1 at day 1, spongiotrophoblast and labyrinthine markers at day 4, and giant cell markers and muc1 at day 7 of differentiation [10], [17], [18 , and data not shown]. For the remaining experiments, these time points were used to determine differences in marker expression, although additional days of differentiation were also frequently examined to avoid misinterpretation due to minor alteration in expression kinetics. Compared to PPARγ^+/+^ TS cells, PPARγ**^−/−^** cells showed increased spongiotrophoblast (Tpbpa) and TGC (Pl-I, Pl-II) differentiation (Figure 5), and decreased labyrinthine/syncytiotrophoblast (Gcm1, syncytin A, muc1, Tfeb) differentiation (Figure 6), by marker analysis.

The striking downregulation of Gcm1 (Figure 6), an early marker and essential determinant of syncytiotrophoblast layer II (SynT-II) differentiation in the placental labyrinth, as well as markers further downstream in labyrinthine/syncytiotrophoblast differentiation (syncytin A, expressed in syncytiotrophoblast layer I/SynT-I labyrinthine cells, and muc1), in the absence of PPARγ, is consistent with the decreased number of multinucleated syncytiotrophoblast in differentiated PPARγ^−/−^ TS cells (Figure 4) [19].

### PPARγ^−/−^ TS Cells Differentiate Preferentially into Invasive Subtypes of Trophoblast Giant Cells

**Figure 7:**
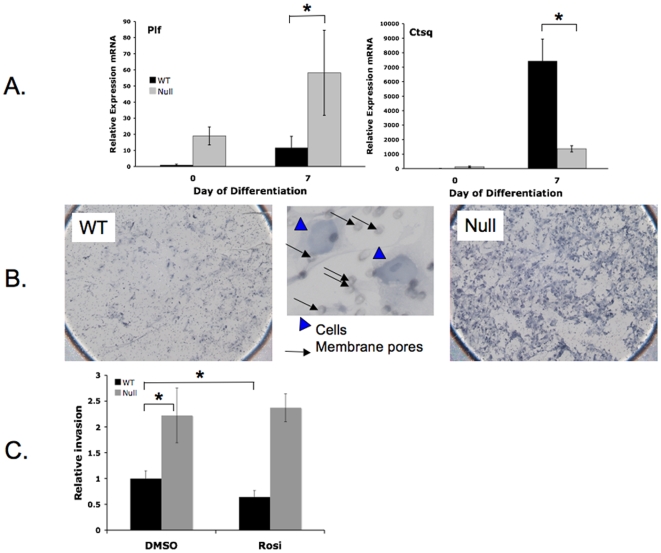
PPARγ^−/−^ TS cells show altered trophoblast giant cell markers and matrix invasion phenotype. A) Plf expression is increased and Ctsq expression is decreased in PPARγ**^−/−^** TS cells. *indicates statistically significant (P≤0.05) alteration in marker expression in null cells, compared to WT cells. B) PPARγ**^−/−^** TS cells, differentiated for 7 days, invade Matrigel in greater numbers, compared to their WT counterparts. Representative images of the underside of Matrigel-coated membrane, following invasion of either WT or PPARγ^−/−^ TS cells are shown; invasive cells show characteristic morphology of TGC (blue arrowheads). Small dots (black arrows) correspond to pores in the membrane. C) Relative invasion of trophoblast in WT and null TS cells with or without rosiglitazone. Note that the effect of rosiglitazone on trophoblast invasion is PPARγ-dependent. *indicates statistically significant (P≤0.05) alteration in invasion, compared to WT cells treated with carrier (DMSO) alone.

Recently, Simmons et al. [20] characterized diverse subtypes of trophoblast giant cells (TGC) based on expression of specific markers, including Pl-I, Pl-II, Plf, and Ctsq, and showed that formation of these subtypes by TS cells is differentially regulated *in vitro*. Specifically, Ctsq is a marker of sinusoidal TGC, likely derived from chorion-proximal Tpbpa^−^ precursors in ectoplacental cone (EPC) and participating in labyrinth formation, while Plf is present in canal-type, spiral artery-associated, and parietal TGC, likely derived primarily from chorion-distal Tpbpa^+^ EPC [19], [20]. In addition to upregulation of Pl-I and Pl-II (30-fold and 3.5-fold, respectively, Figure 5), we also found that Plf is upregulated ∼4-fold while Ctsq is downregulated ∼3-fold in differentiated PPARγ^−/−^ compared to WT TS cells (Figure 7A). This suggests that PPARγ^−/−^ TS cells preferentially differentiate to invasive trophoblast subtypes, including canal-type and parietal TGC, and do not readily form sinusoidal TGC, an important constituent of chorion and fully differentiated labyrinth [19]. To determine whether the functional capacity of PPARγ**^−/−^** TS cells mirrors their altered expression profile of TGC genes, we compared the behavior of PPARγ^+/+^ and PPARγ**^−/−^** TS cells, differentiated for 7 days, in a Matrigel invasion assay. As previously described [21], the majority of cells invading Matrigel were morphologically consistent with TGC (arrowheads in Figure 7B). In addition, although similar numbers of WT and PPARγ**^−/−^** differentiated TGC were applied to the membrane (see Figure 4; ∼92,700 TGC in wild-type vs. ∼95,200 TGC in null cells after 7 days of differentiation), over two-fold more PPARγ^−/−^ TGC invaded to the far side of the Matrigel membrane compared to their WT counterparts (Figure 7C), consistent with an enhanced invasive capacity of individual TGC in the absence of PPARγ.

### Rosiglitazone Inhibits Trophoblast Giant-Cell Differentiation and Invasion in a PPARγ-Dependent Manner

**Figure 8:**
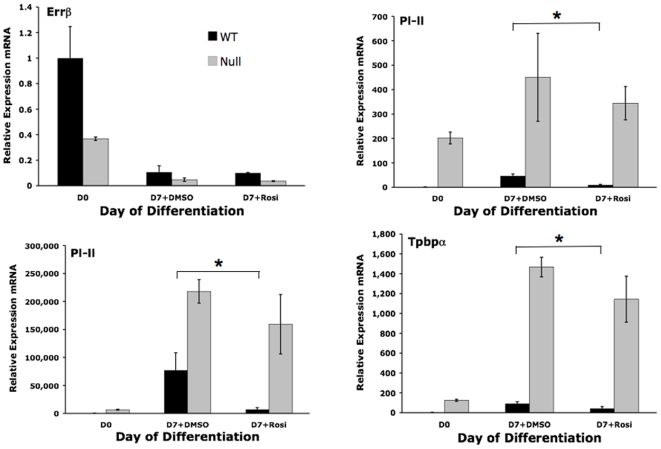
Rosiglitazone, a PPARγ agonist, downregulates giant cell markers in a PPARγ-dependent manner. *indicates statistically significant alteration in marker expression between the indicated conditions.

**Figure 9:**
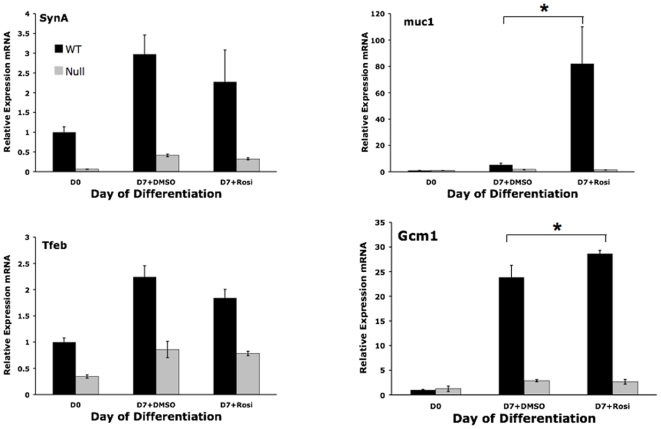
Rosiglitazone, a PPARγ agonist, upregulates select labyrinthine markers in a PPARγ-dependent manner. *indicates statistically significant alteration in marker expression between the indicated conditions.

To determine the effects of pharmacologic activation of PPARγ on trophoblast, we tested the ability of rosiglitazone, a specific PPARγ agonist, to alter TS cell proliferation and differentiation *in vitro*. While TS cell proliferation was not altered (data not shown), expression of differentiation markers (Figures 8 and 9) was differentially regulated by treatment of PPARγ^+/+^ TS cells, but not PPARγ^−/−^ TS cells, with 1 µM rosiglitazone. Specifically, while rosiglitazone decreased expression of the spongiotrophoblast marker Tpbpa (2.2 fold, *P* = 0.0287) and giant cell markers Pl-I (5 fold, *P* = 0.0025) and Pl-II (11 fold, *P* = 0.0183;), the labyrinthine markers syncytin A and Tfeb were not affected (Figures 8 and 9). However, muc1, a known direct transcriptional target of PPARγ in trophoblast [10], was increased 16 fold (*P* = 0.0089) and Gcm1 was increased 1.2 fold (*P* = 0.03) by rosiglitazone (Figure 9). The effects of rosiglitazone were PPARγ-dependent, as all were absent in PPARγ**^−/−^** TS cells (Figures 8 and 9). In addition, downregulation of giant cell markers was not accompanied by changes in the morphologic assessment of differentiation, as the percentage of giant cells and syncytiotrophoblast remained the same with or without rosiglitazone (data not shown). Finally, rosiglitazone decreased invasion of differentiated TS cells by 36% in a PPARγ-dependent manner (Figure 7C; *P* = 0.0342).

### Introducing PPARγ in Genetically Deficient TS Cells Rescues Syncytiotrophoblastic and Labyrinthine Differentiation

Since PPARγ-null TS cells preferentially and prematurely differentiate into TGC at the expense of labyrinthine cell types, and since PPARγ activation by rosiglitazone downregulates markers of TGC while upregulating some labyrinthine genes, we hypothesized that PPARγ promotes labyrinthine/syncytiotrophoblastic differentiation. To directly test this hypothesis, we generated an adenoviral construct expressing a murine PPARγ1-HA fusion protein and co-expressing GFP from an internal ribosome entry site. Western blot verified formation of an appropriately-sized HA-tagged protein (data not shown). PPARγ-null TS cells were infected with the highly purified adenovirus; virus expressing only the backbone GFP construct, without the PPARγ1 gene, was used as control. Both GFP and HA-PPARγ1 were detectable in TS cell cultures seven days after infection, corresponding to day 6 of differentiation.

**Figure 10:**
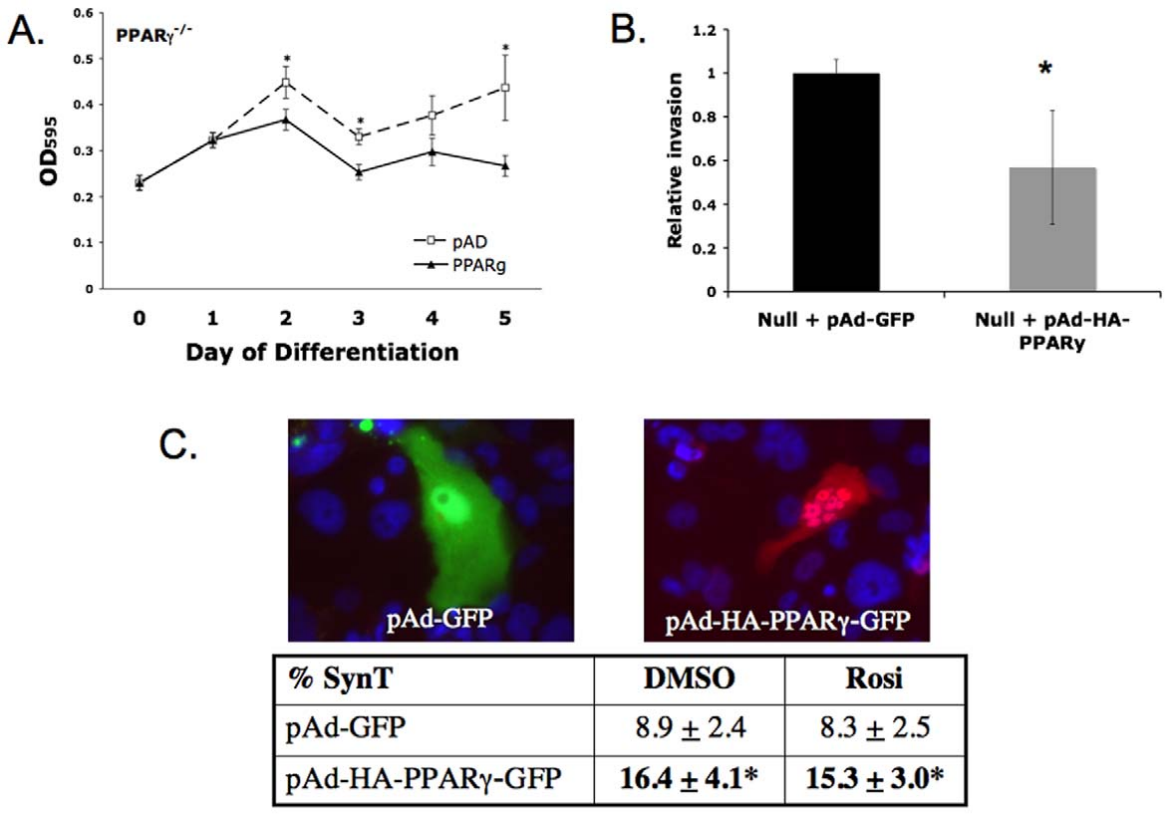
Effects of reintroducing PPARγ into PPARγ^−/−^ TS cells. A) PPARγ overexpression in undifferentiated null TS cells further inhibits cell growth. B) Reintroduction of PPARγ into PPARγ^−/−^ TS cells decreases trophoblast invasion. *P<0.05. C) Morphologic assessment of cellular differentiation in null TS cells infected with either GFP-expressing adenovirus, or adenovirus expressing HA-tagged PPARγ1. Images are representative of cells infected with either GFP-expressing adenovirus (left) or HA-tagged PPARγ (right), with nuclei counterstained with DAPI. Cell counts are from three independent experiments (* *P*<0.05 compared to pAd-GFP-infected cells).

**Figure 11:**
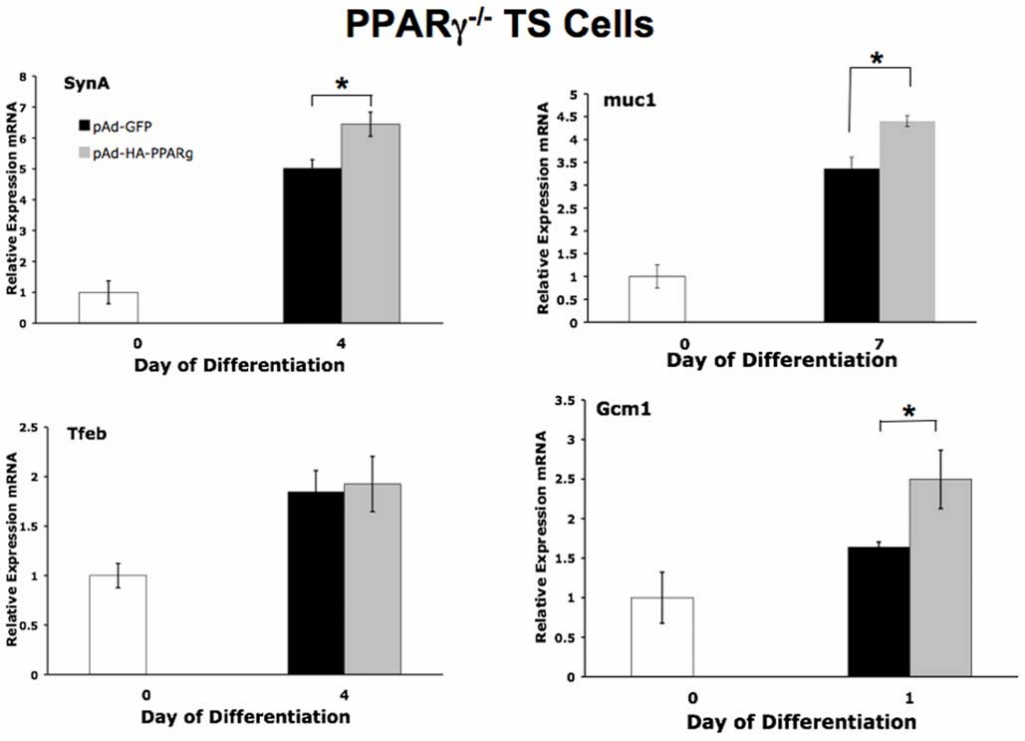
Labyrinthine marker assessment in null TS cells infected with either GFP- or PPARγ-expressing adenovirus. Gcm1, muc1, and SynA, but not Tfeb, are increased in PPARγ-virus-infected null cells. White columns indicate values for an aliquot of the same undifferentiated PPARγ**^−/−^** TS cells used for transduction but not exposed to virus. *indicates *P*<0.05 compared to pAd-GFP-infected cells.

PPARγ-expressing virus slowed growth of PPARγ-null TS cells in TSMFH medium (Figure 10A). Also, reintroduction of PPARγ into null cells decreased Matrigel invasion by 44% (Figure 10B; *P* = 0.0487). When induced to differentiate, PPARγ-virus-infected null cells formed almost twice as many multi-nucleated syncytiotrophoblast, as control virus-infected cells (Figure 10C). In addition, by qRT-PCR analysis, Gcm1, synA, and muc1 expression was induced 1.3–1.5 fold in differentiated PPARγ-virus-infected null TS cells compared to control virus-infected cells (Figure 11, *P*<0.05 for each). In contrast, Tfeb and all giant cell markers were unaffected (Figure 11, and data not shown). Both WT and PPARγ-null TS cells when differentiated in media containing DMSO, the carrier used for rosiglitazone, routinely showed more syncytialized cells than did TS cells cultured without DMSO (compare Figure 10, with DMSO, to Figure 4, without DMSO). The additional presence of PPARγ increased percentage of syncytiotrophoblast well above and beyond this DMSO effect; however, rosiglitazone did not further alter differentiation of PPARγ-virus-infected null TS cells as assessed either by morphology or by qRT-PCR (Figure 10C, and data not shown).

## Discussion

**Figure 12:**
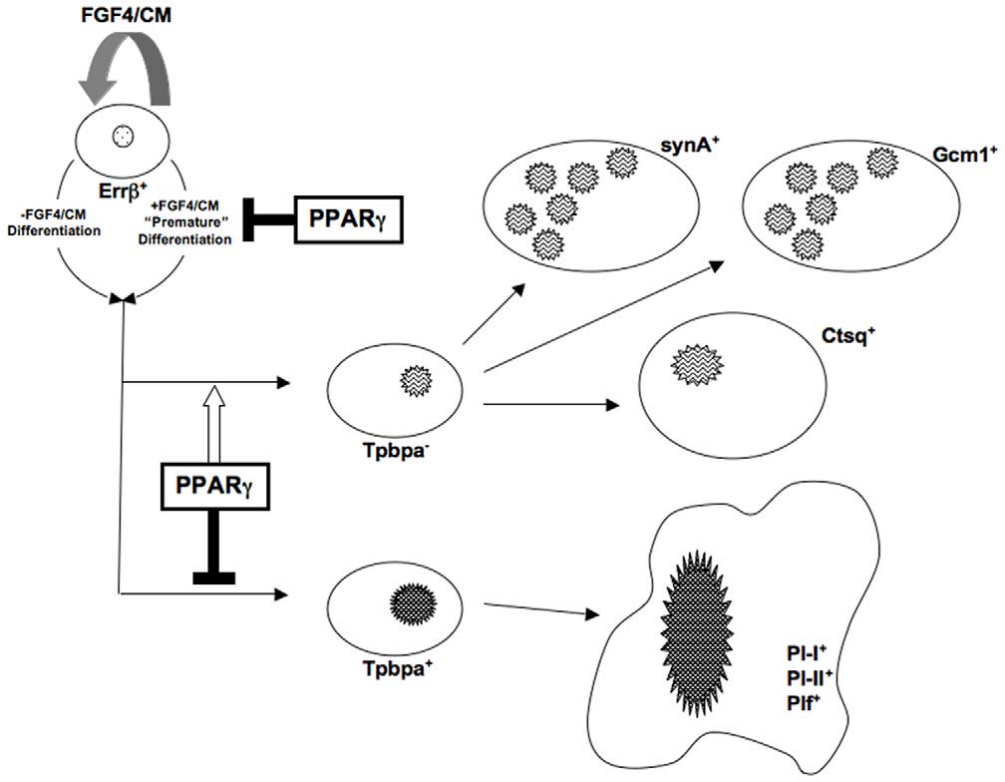
Model of PPARγ's role in trophoblast differentiation. Note that PPARγ contributes to both maintenance of undifferentiated trophoblast (by inhibiting premature differentiation) and differentiation towards the labyrinthine lineages. Errβ^+^ cells denote undifferentiated TS cells; FGF4/CM refers to the presence of FGF4 and MEF-conditioned media, both of which are required for maintenance of the undifferentiated state. The spongiotrophoblast (Tpbpa^+^ giant cell precursor) and trophoblast giant cell (Pl-I^+^, Pl-II^+^, Plf^+^) lineages are distinguished from labyrinthine precursor (Tpbpa^−^) cells as well as fully differentiated labyrinthine cells (synA^+^ SynT-I, Gcm1^+^ SynT-II, and Ctsq^+^ mononuclear cells).

PPARγ is expressed at high levels in all placental layers of the mouse, starting as early as E8.5 (8.5 days post-coitum) [6 ; Milstone et al., manuscript in preparation]; however, its role in murine trophoblast differentiation has not been extensively studied. PPARγ-null mice die at midgestation (E10.5-E11.5) due to abnormalities of the placenta, characterized by small labyrinth, reduced spongiotrophoblast, and expanded giant cell layers [6], [7]. To better understand the role of PPARγ in placental development and function, we generated both wild-type and PPARγ-null trophoblast stem (TS) cells and characterized cellular properties *in vitro*. We determined that PPARγ-null TS cells show defective differentiation of all three lineages contributing to labyrinth [19], with decreased expression of Ctsq (marking sinusoidal TGC after E12.5), syncytin A (marking syncytiotrophoblast layer I/SynT-I), and Gcm1 (marking syncytiotrophoblast layer II/SynT-II) (Figures 6 and 7A). Instead of forming labyrinth, PPARγ-null TS cells differentiate prematurely and preferentially into trophoblast giant cells. These observations are consistent with the effects of PPARγ genetic deficiency on placental development *in vivo*, particularly the extremely small labyrinth [6], [7], and suggest that PPARγ contributes directly to both maintenance of undifferentiated trophoblast and differentiation of the labyrinthine pathway. These results are summarized in the model of PPARγ action in trophoblast differentiation presented in Figure 12.

Muc1 and PGAR (PPARγ angiopoietin related/FIAF/ANGPTL4) are the only known direct transcriptional targets of PPARγ in placenta [10], [22]. Our data show that Gcm1 mRNA expression is induced both by rosiglitazone treatment of WT (but not null) TS cells (1.2 fold increase, Figure 9) and by reintroduction of PPARγ into null TS cells (1.5 fold increase, Figure 11). This induction may represent direct transcriptional regulation by PPARγ, as the proximal Gcm1 promoter contains at least one putative “atypical” PPARγ-response element (PPRE) [23], [24 , and data not shown]. Luciferase reporter gene assays are currently in progress to test PPARγ1 regulation of transcriptional initiation at the proximal murine Gcm1 promoter [23].

The effect of rosiglitazone on downregulation of spongiotrophoblast/giant cell markers is consistent with previously published *in vivo* data, showing that treatment of pregnant mice with rosiglitazone downregulates placental Tpbpa expression and reduces the spongiotrophoblast compartment [11]. We also observed that rosiglitazone upregulates differentiated TS cell expression of muc1 (Figure 9), a known direct transcriptional target of PPARγ in labyrinthine trophoblast *in vivo* [10]. However, while it induced Gcm1 expression, rosiglitazone did not alter the additional labyrinthine markers syncytin A or Tfeb, or cause morphologic evidence of syncytiotrophoblastic differentiation in our experiments (Figures 9, 10C, and data not shown). This induction of some but not all mediators and consequences of syncytiotrophoblast formation and labyrinthine differentiation, processes we hypothesize are PPARγ-dependent, could reflect near-maximal PPARγ activation by mediators already present in differentiating TS cell cultures and/or different roles of PPARγ in formation of SynT-I and SynT-II cells. In addition, although wild type TS cells contribute robustly to all lineages *in vivo*, they show limited (only 5–10%) formation of labyrinthine cell types under the “standard” *in vitro* differentiation conditions [14], [18 , and this paper], [Figure 4]. In contrast, HIF1β/ARNT-deficient TS cells express high levels of Gcm1 and preferentially differentiate to syncytiotrophoblast *in vitro* [17]. Interestingly, differentiated HIF1β/ARNT^−/−^ TS cells also contain high levels of acetylated histone-4, and inhibition of histone deacetylase in wild type TS cells phenocopies the HIF1β/ARNT^−/−^ mutation [17]. These data suggest that the Gcm1 promoter may be less accessible to transacting factors in cultured TS cells, due to a condensed chromatin structure, thereby limiting induction by potential transcriptional regulators, such as PPARγ. Epigenetic effects may thus play a prominent role in PPARγ-dependent trophoblast differentiation and placental development *in vivo* but may be incompletely recapitulated by TS cells differentiating *in vitro*.

Differentiated PPARγ^−/−^ TS cells showed a 36% decrease in syncytiotrophoblast, assessed morphologically, compared to WT cells (4.7% compared to 7.3%; Figure 4). Syncytiotrophoblast differentiation was also restored (increased 84%) in PPARγ**^−/−^** TS cells by reintroducing PPARγ using adenovirus, and was not further altered by rosiglitazone (Figure 10). However, restoring PPARγ expression in null cells only modestly altered labyrinthine marker expression (Figure 11) and did not alter giant cell markers (data not shown). This apparent quantitative discrepancy between morphologic and molecular differentiation may reflect, in part, that only 20–50% of PPARγ**^−/−^** TS cells were infected by adenovirus (determined by GFP fluorescence), even at the highest MOI, and only 50% of these infected cells also showed nuclear localization of exogenous PPARγ (determined by immunofluorescent detection of HA tag) (Figure 10C and data not shown). Because syncytiotrophoblast cells were reported as a percent of the cells showing nuclear localized exogenous PPARγ, while mRNA was (necessarily) quantified in the entire culture, the expression data underestimates the functional effects of exogenous PPARγ, and the magnitude of the discrepancy is proportional to the fraction of uninfected cells. In fact, if all infected cells were counted (those expressing HA-PPARγ in the nucleus, the cytoplasm or both), the percentage of syncytiotrophoblast increased from 8.3% to 11.5% (38% increase), not to 15.3% (84% increase). However, assessed either way, the effect of exogenous PPARγ on syncytiotrophoblast differentiation remained statistically significant (*P*<0.05). In addition, nuclear HA-PPARγ was present in all syncytiotrophoblast differentiated from PPARγ**^−/−^** TS cells rescued by Ad-HA-PPARγ-GFP. This suggests that PPARγ supports syncytiotrophoblast differentiation by regulating activity of a nuclear/transcription factor(s). This could include upregulation of a factor inducing labyrinthine differentiation (such as Gcm1) or inhibition of factors favoring the spongiotrophoblast/giant cell lineage. Finally, enhanced giant cell differentiation of PPARγ^−/−^TS cells may also, in part reflect unopposed action, and perhaps upregulation of PPARβ/δ (see Figure 1D), which is known to induce giant cell differentiation both *in vivo* and *in vitro* [25].

PPARγ**^−/−^** TS cells grew more slowly and showed enhanced TGC, but not syncytiotrophoblast differentiation in TSMFH medium relative to their WT counterparts. This suggests that PPARγ^−/−^ TSC preferentially differentiate to TGC even under stem cell conditions (in TSMFH media). Unlike its effect on syncytiotrophoblast differentiation, reintroducing PPARγ into null cells exacerbated, rather than reversed, the growth abnormality (Figure 10A). This divergent behavior might reflect differential sensitivity of growth and differentiation to PPARγ activity. CMV promoter-driven expression from Ad-HA-PPARγ-GFP was 12-fold higher than endogenous PPARγ levels observed in WT TS cell cultures, and was equivalent to levels normally achieved only after 4 days of differentiation (Figure 1C and data not shown). This suggests that low level PPARγ expression stabilizes the TS cell undifferentiated state under growth conditions, while high level expression favors syncytiotrophoblast formation when differentiation conditions predominate (Figure 12); artificially imposing high level expression in the absence of differentiation conditions may suppress TS cell proliferation directly.

In contrast to these defects in labyrinthine trophoblast development, elevated expression of Plf, Pl-I, and Pl-II, suggests enhanced differentiation of PPARγ^−/−^ TS cells into parietal, canal and spiral artery associated TGCs [19], each of which are involved in establishing the maternal-fetal interface, analogous to invasive-type trophoblast in human placenta. Because PPARγ has been implicated in human trophoblast invasion [8], we analyzed PPARγ-null TS cells in Matrigel invasion assays (Figure 7B). PPARγ-null TGC showed enhanced trophoblast invasion, on a per cell basis, which was reversed by reintroduction of PPARγ (Figure 10B). In addition, rosiglitazone inhibited trophoblast invasion, in a PPARγ-dependent manner (Figure 7C). This data is consistent with previous studies showing inhibition of human cytotrophoblast invasion by PPARγ agonists [9].

In summary, the characterization of PPARγ-null TS cells has shed more light on the role of PPARγ in trophoblast differentiation and placental development. The data are consistent with the *in vivo* defects of PPARγ-deficient placentas and confirm the utility of cultured TS cells in studies of placental development. We have shown that PPARγ regulates trophoblast proliferation, invasion, and labyrinthine differentiation, and we hypothesize that these effects are mediated, at least partially, through Gcm1. More extensive gene expression analysis, using whole mouse genome expression arrays, will further identify potential downstream targets of PPARγ in trophoblast differentiation. These studies will help elucidate the role of PPARγ in placental development and determine the utility of specific agonists in treatment of placenta-related pregnancy disorders.

## Materials and Methods

### Ethics Statement

All animal work was approved by, and conducted according to the guidelines of, the Institutional Animal Care and Use Committee at Brigham and Women's Hospital.

### Derivation and Culture of TS Cells

PPARγ^+/+^ and PPARγ^−/−^ TS cells were derived from E3.5 blastocysts from PPARγ^+/−^×PPARγ^+/−^ matings of mice with mixed (C57Bl/6J:129:FVB) genetic background [26], [27], on murine embryonic feeder cells (MEF), and cultured as previously described [14]. For feeder-free cultures, TS cells without MEF were grown in 30% TS cell medium (TS medium), 70% MEF-conditioned (72 h) TS medium, 25 µg/ml FGF4 (Sigma), and 1 µg/ml heparin (Sigma) (TSMFH medium). TS cells grown to confluence in TSMFH medium were switched to TS medium to induce differentiation. All cells were cultured at 37°C under 95% room air and 5% CO_2_.

### Morphologic Analysis of Differentiation

TS cells were cultured as described above, on glass coverslips. Cells were fixed in 2% paraformaldehyde in PBS for 15 minutes and permeabilized in 0.02% Triton X-100 in PBS for 2 minutes. Mouse anti-HA tag antibody (Abcam) and Alexa dye-conjugated phalloidin and secondary antibodies (Molecular Probes) were used per manufacturer's instructions. Coverslips were mounted using Vectashield mounting media containing DAPI (Vector labs). Trophoblast giant cells were identified by large single or double nuclei, each at least twice the size (judged as estimated cross-sectional area) of an undifferentiated TS cell nucleus; syncytiotrophoblast were identified as cells with 3 or more nuclei, each of which were similar in size to an undifferentiated TS cell nucleus.

### Proliferation and Invasion Assays

Cell proliferation was assayed using the MTT Cell Proliferation Kit I (Roche), with cells plated in 96-well plates at an initial density of 5×10^3^ cells per well. Five replicate wells were assayed for each condition and/or time point.

Matrigel invasion assays were done as described previously [21] with the following modifications. Cells were differentiated for 7 days in TS media; on day 7, cells were trypsinized and plated onto the Matrigel at a concentration of 10^5^ cells per insert. The media in the lower chamber was changed every day. After 3 days, the Transwell inserts were fixed for 15 minutes with 3.7% formaldehyde in PBS and washed with PBS. Cells that remained at the upper surface of the filters were scraped off and filters were stained overnight with hematoxylin. The remaining cells (migrated to the bottom of the filter) were imaged and counted using a light microscope (Leica).

### RNA Isolation and Quantitative Reverse Transcription-PCR (qRT-PCR)

DNase-treated total RNA was isolated using NucleoSpin RNA II kit (Clontech). cDNA was prepared from 1 µg RNA using iScript (Bio-Rad) in a 20 µl reaction, and diluted 1∶5 with nuclease-free water. PCR was performed using 4 µl of the diluted cDNA, along with 625 nM of each primer and POWER SYBR Green PCR master mix (Applied Biosystems) in a total reaction volume of 20 µl. qRT-PCR was performed using a System 7300 instrument (Applied Biosystems) and a one-step program: 95°C, 10 min; 95°C, 30 s, 60°C, 1 min, for 40 cycles. Unless otherwise stated, each experiment was performed in triplicate, and all results were normalized against 18S rRNA. Relative mRNA expression levels, compared to 18S rRNA, were determined by the ΔΔC_T_ method [28]. Fold change in normalized expression of individual genes in experimental samples was determined by comparison to expression in undifferentiated PPARγ^+/+^ cells. All primer pairs (Table 1) were checked for specificity using BLAST analysis and were checked by both agarose gel electrophoresis and thermal dissociation curves to ensure amplification of a single product with the appropriate size and melting temperature.

### Whole Cell Lysate Preparation and Western Blotting

Cells were washed with cold 1X PBS and lysates were prepared by scraping cells in boiled 1X Laemmli sample buffer (Bio-Rad), and passing the lysate through a 25G needle to sheer DNA and decrease viscosity. Protein determination was done using a Coomassie-binding assay [29]. For Western blot, 20 µg of total protein was loaded in each lane. After transfer to PVDF, the membranes were blocked with 5% non-fat dried milk in TBS-Tween for 1 hour at room temperature. Primary and secondary antibody incubations were similarly performed, with antibodies diluted in blocking buffer per the manufacturer's recommendation. Antibodies used included mouse anti-PPARγ monoclonal antibody and rabbit polyclonal antibodies against PPARα and PPARβ (all three from Santa Cruz Biotechnology, Inc.), mouse anti-actin monoclonal antibody (Abcam) used as loading control, and HRP-conjugated secondary antibodies (Jackson Immunochemicals). After treatment with ECL reagent (Pierce), membranes were exposed to autoradiography film (Kodak). Expression was quantified by densitometric scanning followed by normalizing PPARγ, PPARα and PPARβ/δ expression to that of β-actin.

### Generation of Adenovirus and Transduction of TS Cells

Full length murine PPARγ1 cDNA was obtained by RT-PCR from E10.5 placental RNA, confirmed by sequence analysis, and cloned into pShuttle-IRES-hrGFP-2 adenoviral vector (Stratagene), in order to generate a hemagglutinin (HA)-tagged protein (HA-PPARγ) co-expressed with GFP. This construct was used to generate adenovirus using the AdEasy XL system (Invitrogen). Large-scale production of adenovirus was accomplished by infecting subconfluent 293A cells (Invitrogen) with the crude viral lysate in 15-cm tissue culture dishes. Virus was purified by CsCl gradient centrifugation, desalted by chromatography on PD-10 columns (Amersham), and stored in 10% glycerol in PBS at −80°C. Titers were determined by plaque assay using 293A cells, according to Invitrogen's Virapower Adenoviral Expression System protocol. TS cells were infected with this purified virus, at an MOI of 10–50, in growth media (TSMFH media: 70% MEF-conditioned medium, with FGF4 and heparin); the day after infection, cells were switched to differentiation media (TS media only) and cultured an additional 1–7 days before harvesting. Expression of HA-PPARγ from this adenovirus was confirmed by both immunofluorescence and Western blot using an anti-HA tag antibody (Abcam). On average, this protocol resulted in infection of 20–50% of TS cells.

### Statistical Analysis

Unless otherwise stated, data presented are mean ± standard deviation of three replicate wells of a single prep of cells; the results shown are representative of 3–5 separate experiments each performed on different days and using a different preparation of cells. Student's t-test was performed and a P value of 0.05 was taken to indicate a statistically significant difference between the populations sampled. Standard deviations are illustrated in all figures, but in some cases are too small to observe.
